# Exploring phosphoregulation of MYO3A using quantitative fluorescence image analysis in COS7 cells

**DOI:** 10.17912/micropub.biology.002189

**Published:** 2026-07-26

**Authors:** Vu M.N. Phan, Omar Alberto Quintero-Carmona

**Affiliations:** 1 Department of Biology, University of Richmond, Richmond, Virginia, United States

## Abstract

Myosin IIIA is an unconventional myosin that contains a kinase domain, and is involved in the formation of hair-cell stereocilia. To investigate its regulatory roles, we mimicked phosphorylation in mchr-MYO3AΔK constructs and assayed their ability to influence filopodial properties in COS7 cells. The phosphomimics generated fewer filopodia. Coexpression of mchr-MYO3AΔK with a GFP-construct containing only the MYO3A kinase domain also resulted in generation of fewer filopodia. Structural predictions suggest that the phosphorylation sites inhibit actin/MYO3A interactions. Taken together, these analyses link MYO3A phosphorylation with the regulation of its ability to create actin protrusions such as filopodia and stereocilia.

**Figure 1. Mimicking MYO3A motor phosphorylation alters COS7 cell filopodia properties:** **(A)** A domain schematic of the constructs used in this study, where the size of the represented MYO3A domains is proportional to the number of amino acids in each region. Human MYO3A consists of a kinase domain (aa1-340), motor domain (aa352-1041), IQ motifs (aa1055-1375), tail homology domain I (THDI, aa1492-1553) and tail homology domain II (THDII, aa1595-1616). **(B)** Representative image of a range of filopodial tip-to-cell-body ratios (TCBR) in a cell expressing mchr-MYO3AΔK. Image is shown using the “Fire” lookup table in FIJI. **(C)** While coexpression of GFP-MYO3A ^FL^ with mchr-MYO3AΔK displayed decreased TCBR, mchr-MYO3AΔK ^T908D^ did not show a decreased TCBR. **(D)** Coexpression of GFP-MYO3A ^FL^ with mchr-MYO3AΔK ^T919D^ also displayed decreased TCBR. **(E)** Expression of either GFP-MYO3A ^FL^ or GFP-MYO3A ^KIN^ with mchr-MYO3AΔK resulted in a decreased TCBR. **(F)** Examples of cells displaying a higher (mchr-MYO3AΔK) and lower (mchr-MYO3AΔK coexpressed with GFP-MYO3A ^FL^ ) filopodia edge density. **(G)** Coexpression of GFP-MYO3A ^FL^ with mchr-MYO3AΔK resulted in decreased filopodia edge density, as did mchr-MYO3AΔK ^T908D^ expression, compared to cells expressing mchr-MYO3AΔK. **(H)** Coexpression of GFP-MYO3A ^FL^ with mchr-MYO3AΔK resulted in decreased filopodia edge density, as did mchr-MYO3AΔK ^T919D^ expression, compared to cells expressing mchr-MYO3AΔK. **(I)** Expression of either GFP-MYO3A ^FL^ or GFP-MYO3A ^KIN^ with mchr-MYO3AΔK resulted in decreased filopodia edge density compared to cells expressing mchr-MYO3AΔK alone. **(J)** Phyre2 modeling of the MYO3A/actin interface predicts that T908 and T919 (yellow) residues are in close proximity to D24 and D25 on actin (green). Addition of negative charges to T908 or T919 via phosphorylation would lead to weakened actin affinity and decreased MYO3A motor activity. **(K)** We propose a model where MYO3A kinase autophosphorylation modulates MYO3A-mediated filopodia initiation. Asterisks indicate p<0.05, Tukey analysis. In the graphs, each marker represents a "biological replicate" consisting of multiple cells, as defined in the "Quantification and statistics" section of the Methods.

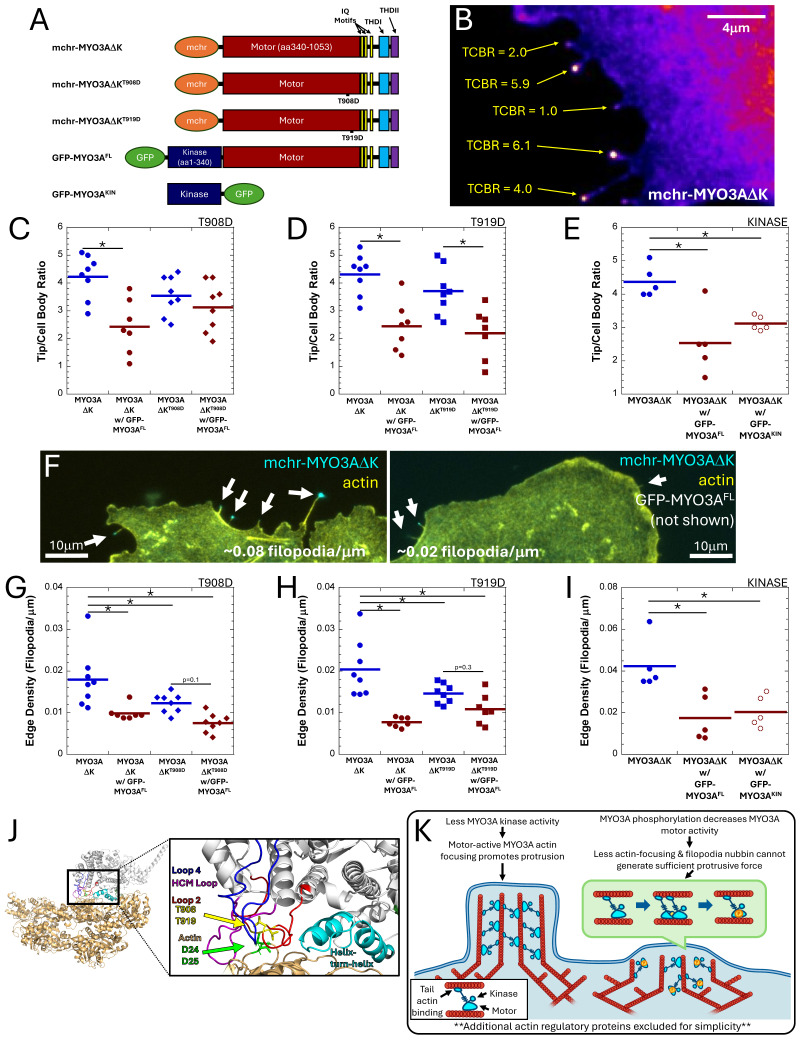



## Description

Class III myosins are a key component of actin-based protrusions known as stereocilia. These structures are found in sensory epithelia, such as the inner ear. Initially characterized in *Drosophila melanogaster* and named NINAC (Montell & Rubin, 1988) *,* class III myosins contain a conserved N-terminal kinase domain (Dosé et al., 2007) proposed to regulate the protein through autophosphorylation (Ng et al., 1996; Quintero et al., 2010, 2013). Two vertebrate isoforms (MYO3A and MYO3B) both localize to the tips of stereocilia (Dosé et al., 2003), and bind to stereocilia resident proteins like the ESPN1 (Merritt et al., 2012; Salles et al., 2009), MORN4 (Mecklenburg et al., 2015), and actin itself (Les Erickson et al., 2003). MYO3A is hypothesized to transport proteins along stereocilia and regulate stereocilia lengths (Dosé et al., 2003). Function-altering mutations in the *MYO3A* gene have been linked to nonsyndromic deafness DFNB30 (T. Walsh et al., 2002; Cirilo et al., 2024). Mouse lines modeling the DFNB30 mutation display age-dependent hearing loss due to degeneration of hair cell stereocilia (V. L. Walsh et al., 2011).

Immunostaining of MYO3A shows strong labeling of the tips of stereocilia and GFP-MYO3A constructs also show strong tip-localization (Schneider et al., 2006). Fluorescently-tagged MYO3A constructs localize to the tips of other actin-based protrusions such as filopodia (Les Erickson et al., 2003; Schneider et al., 2006) and microvilli (An et al., 2014; Raval et al., 2016) in multiple cultured cell types. Kinase domain deletion (MYO3AΔK) led to enhanced tip-localization of GFP-MYO3AΔK constructs in both hair cell stereocilia (Schneider et al., 2006) and COS7 cell filopodia (Salles et al., 2009). Additionally, introducing a kinase-inactivating mutation into full length GFP-MYO3A still led to tip localization in stereocilia and also led to enhanced tip localization in COS7 cell filopodia (Quintero et al., 2010). Ectopic expression of fluorescently-tagged MYO3AΔK constructs in COS7 cells results in an increase in the number of filopodia extending from the edge of the cell (Quintero et al., 2010). The MYO3A kinase domain is capable of intermolecular autophosphorylation *in vitro* (Komaba et al., 2003, 2010) and in cultured cells (Quintero et al., 2010) *.* When kinase-functional GFP-MYO3A ^FL^ constructs are coexpressed with kinase-deleted mchr-MYO3AΔK constructs, the filopodial initiation phenotype is minimized (Quintero et al., 2010). One current model for MYO3A function imagines that autophosphorylation of the kinase domain happens at the tips of actin protrusions and controls the available concentration of MYO3A in that compartment by negatively influencing activity via additional autophosphorylation of the motor (Quintero et al., 2013).

Phosphoproteomic analysis of *in vitro* phosphorylated human MYO3A identified a number of putative phosphorylation sites—T184 in the kinase domain, and T908 & T919 in the motor domain (Quintero et al., 2013). The web resource, Phosphosite Plus, lists 18 additional putative phosphorylation sites spread across the entirety of the protein (Hornbeck et al., 2015). Although the consequences of phosphorylation of the MYO3A motor domain have been studied using *in vitro* steady-state assays and cell-based assays (Komaba et al., 2010; Quintero et al., 2013), the potential biological function of specific phosphorylation at T908 or T919 has yet to be investigated. To do so, we generated point mutations in mchr-MYO3AΔK construct to mimic phosphorylation, replacing threonine with aspartic acid (mchr-MYO3AΔK ^T908D^ and mchr-MYO3AΔK ^T919D^ , Figure 1A ), and applied quantitative imaging techniques to elucidate the biological significance of such posttranslational modifications on actin protrusion-related functions of MYO3A. For the following experiments, the data represent multiple biological replicates or "n." The number of cells and filopodia described by a replicate depend on experimental approach as defined in the Methods section, "Quantification and statistics."

We assayed phosphomimic constructs for their ability to tip-localize in COS7 cell filopodia ( Figure 1B ), which do not express MYO3A endogenously and do not generate many filopodia. There was no difference in the tip to cell body ratio (TCBR) between cells expressing mchr-MYO3AΔK ^T908D^ (3.6±0.3, n=8) compared to mchr-MYO3AΔK (4.2±0.3, n=8, Figure 1C ). Cotransfection of the mchr-constructs with GFP-MYO3A ^FL^ decreased the TCBR for cells coexpressing mchr-MYO3AΔK (2.4±0.4, n=7, p<0.001, Tukey analysis vs mchr-MYO3AΔK alone) but not for cells coexpressing mchr-MYO3AΔK ^T908D^ (3.1±0.3, n=8, p=0.06, Tukey analysis vs mchr-MYO3AΔK ^T908D^ alone, Figure 1C ). However, cells expressing mchr-MYO3AΔK ^T919D^ in combination with GFP-MYO3A ^FL^ (2.2±0.3, n=8) showed a decrease in TCBR compared to cells expressing mchr-MYO3AΔK ^T919D^ alone (3.7±0.3, n=8, p<0.01, Tukey analysis, Figure 1D ). The differential behavior of the two phosphomimics could be due to biologically relevant differences in the impact of T908 phosphorylation versus T919 phosphorylation on MYO3A motor activity. Alternatively, the T to D substitution may be a poorer mimic of phosphorylation at T908 than at T919. It is also possible that, similar to actin polymerization, MYO3A-mediated filopodia initiation requires MYO3A concentrations above a critical concentration, so impacts on the amount of MYO3A at filopodia tips would be difficult to visualize in existing filopodia. In this instance, the impacts of phosphomimicry might be visible by measuring the number of filopodia generated by the cell.

We then assayed the phosphomimic constructs for their ability to influence filopodial edge density ( Figure 1F ). There was a significant decrease in the filopodia edge density for cells expressing mchr-MYO3AΔK ^T908D^ (0.012±0.001 filopodia/μm, n=8) compared to cells expressing mchr-MYO3AΔK (0.018±0.002 filopodia/μm, n=8, p<0.04, Tukey analysis, Figure 1G ). Coexpression of the mchr-constructs with kinase-functional GFP-MYO3A ^FL^ resulted in a statistically significant decrease in filopodial edge density for cells expressing mchr-MYO3AΔK (0.010± 0.001 filopodia/μm, n=8, p<0.003, Tukey analysis vs mchr-MYO3AΔK alone) but not for cells expressing mchr-MYO3AΔK ^T908D^ (0.008±0.001 filopodia/μm, n=8, p=0.1, Tukey analysis vs mchr-MYO3AΔK ^T908D^ alone, Figure 1G ). The same pattern was observed when comparing cells expressing mchr-MYO3AΔK (0.020±0.002 filopodia/μm, n=8), mchr-MYO3AΔK ^T919D^ (0.015±0.001 filopodia/μm, n=8, p<0.05, Tukey analysis vs mchr-MYO3AΔK alone), and mchr-MYO3AΔK coexpressed with GFP-MYO3A ^FL^ (0.008±0.0004 filopodia/μm, n=7, p<0.0001, Tukey analysis vs mchr-MYO3AΔK alone, Figure 1H ). Coexpression of mchr-MYO3AΔK ^T919D^ with GFP-MYO3A ^FL^ did not significantly decrease filopodial edge density compared to cells expressing mchr-MYO3AΔK ^T919D^ alone (0.011±0.001 filopodia/μm, n=7, p=0.3, Tukey analysis, Figure 1H ). Taken together, these data suggest that phosphoregulation may have related-but-distinct impacts depending on the specific residue. Overall, phosphorylation of the MYO3A motor domain influences the ability of MYO3A-expressing cells to maintain actin protrusions by either stabilizing already-formed protrusions, or by stimulating protrusion initiation.

If MYO3A autophosphorylation is negatively-regulating protrusion formation activity, then autophosphoregulation might be occurring at filopodial initiation sites on the inner surface of the plasma membrane. Such phosphorylation would not require MYO3A localization to tips of existing filopodia in order to autophosphorylate MYO3A molecules, and MYO3A kinase-alone truncations are capable of phosphorylating MYO3AΔK constructs in *in vitro* assays (Komaba et al., 2010; Quintero et al., 2010). To test the hypothesis that MYO3A phosphoregulation does not require motor activity to influence protrusion initiation, we transfected COS7 cells with mchr-MYO3AΔK in combination with a kinase-domain-only truncation of MYO3A (GFP-MYO3A ^KIN^ ). Unlike MYO3A constructs that contain an active motor domain (Quintero et al., 2010), GFP-MYO3A ^KIN^ does not concentrate at filopodial tips. Cotransfection of either GFP-MYO3A ^FL^ (2.5±0.4, n=5, p<0.002, Tukey analysis) or GFP-MYO3A ^KIN^ (3.1±0.1, n=5, p<0.02, Tukey analysis) with mchr-MYO3AΔK resulted in significant decrease in TCBR compared to mchr-MYO3AΔK alone (4.4±0.2, n=5, Figure 1E ). Cotransfection of either GFP-MYO3A ^FL^ (0.017±0.005 filopodia/μm, n=5, p<0.007, Tukey analysis) or GFP-MYO3A ^KIN^ (0.021±0.003 filopodia/μm, n=5, p<0.02, Tukey analysis) with mchr-MYO3AΔK also resulted in significant decrease in filopodia edge density compared to mchr-MYO3AΔK (0.042±0.005 filopodia/μm, n=5, Figure 1I ). This implies that intermolecular phosphorylation can occur in cells independently of motor function, and in other compartments besides the tips of actin protrusions. Such phosphoregulation could control the density of actin protrusion initiation events by regulating the amount of active MYO3A at filopodial initiation patches on the inner surface of the plasma membrane.

To better understand the impact of T908 and T919 phosphorylation on actin/MYO3A interactions, we threaded the human MYO3A sequence to a model structure for the actomyosin interface (Lorenz & Holmes, 2010). Actomyosin interactions are partially dependent on electrostatic interactions between the two molecules, and this model predicted that both phosphorylation sites are likely positioned in close proximity to acidic patches on actin containing aspartic acid residues D24 and D25 ( Figure 1J ). Phosphorylation would then introduce negative charge near acidic residues, decreasing MYO3A’s affinity for actin, negatively influencing motor activity, and negatively impacting actin protrusion initiation. Previous *in vitro* studies of the MYO3A motor revealed that phosphorylation decreased steady-state actin affinity (K _actin_ ), decreased maximal ATPase rate (k _cat_ ) and increased actin concentration required for half-maximal ATPase rate (K _ATPase_ )--all indicators of decreased myosin function (Dosé et al., 2008; Komaba et al., 2010).

So far, we have only determined the individual effects of phosphorylation at T908 and T919, and accumulation of phosphorylations could have additive effects. Future assays could determine whether double phosphorylation results in stronger inactivation of MYO3A filopodial initiation activity. Additionally, the generation of individual phosphonulls (T908A, T919A, and the double-phosphonull) could reveal whether these or other sites are responsible for regulating additional MYO3A activities.

Since intermolecular autophosphorylation can occur in the absence of MYO3A motor activity, MYO3A kinase activity could regulate actin-based protrusion initiation by MYO3A at nascent filopodial initiation sites along the plasma membrane. Where initial models of MYO3A phosphoregulation described activity at the tips of existing actin protrusions, we propose a refinement of the model where the amount of MYO3A available at protrusion initiation sites is determined by concentration-dependent MYO3A autophosphorylation prior to extension of a protrusion and its corresponding tip ( Figure 1K ). As active, unphosphorylated MYO3A collecting in patches at the membrane would induce actin protrusions, and as the local MYO3A concentration increased, MYO3A kinase activity would limit the concentration of active MYO3A available at the membrane to initiate protrusion. Such a mechanism could regulate the size of protrusion initiation assemblies, influencing the density of actin-based protrusions at the periphery. Overexpression of human MYO3AΔK elongated hair cell stereocilia, and displayed atypical “floppiness” (Schneider et al., 2006). Elimination of kinase activity resulted in increased actin protrusions in cells that generate filopodia (Quintero et al., 2010, 2013) and microvilli (Raval et al., 2016). In this way, the presence of kinase activity could tune the motor domain’s activity, controlling the cell's ability to generate actin protrusions.

## Methods

*Expression Plasmids*

mchr-MYO3AΔK, GFP-MYO3A ^FL^ plasmids had been generated previously (Quintero et al., 2010). The phosphomimic mchr-MYO3AΔK ^T908D^ and mchr-MYO3AΔK ^T919D^ constructs were generated by performing site-directed mutagenesis on the mchr-MYO3AΔK construct. The GFP-MYO3A ^KIN^ construct was generated using megaprimer PCR mutagenesis (Attwell et al., 2003) to move the human MYO3A kinase domain (amino acids 1-351) into pEGFP-N1 (Clontech). All expression plasmids were sequence-verified. A schematic of these constructs can be found in Figure 1A .

*COS7 cell culture and transient transfection*

COS7 (Gluzman, 1981) cells were cultured in growth media: DMEM high glucose (Gibco) that was supplemented with 10% Fetal Bovine Serum (Benchmark FBS, GeminiBio), and antibiotics (50U/mL penicillin, 50µg/mL streptomycin, Gibco). Cells were kept in a humidified incubator at 37°C, with a 5% CO _2_ atmosphere, and passaged using 0.25% trypsin-EDTA (Gibco). In preparation for transfection, cells were plated onto acid-washed, 22mm ^2^ , #1.5 coverslips at a concentration of ~30,000 cells/coverslip (one coverslip per well in a six well dish) and then allowed to adhere overnight. Cells were transfected using Lipofectamine 3000, according to manufacturer’s protocol. For each sample well, 0.3µg of plasmid DNA was diluted into 125µL of Opti-MEM media (Invitrogen) without serum or antibiotics and mixed with 3µL of P3000 reagent. In a separate tube, 4µL of L3000 reagent was diluted into 125µL Opti-MEM. The two tubes were then combined, vortexed and incubated at room temperature for 15 minutes prior to dropwise addition to the sample well. Following transfection, the cells were allowed to grow overnight (16-24h) prior to fixation and counterstaining. For co-transfections, a total of 0.6µg of plasmid DNA is used instead (0.3µg of each plasmid).

*Fixed cell sample preparation and imaging*

Samples were fixed for 20 minutes in PBS containing 4% paraformaldehyde, permeabilized for 5 mins in PBS containing 0.5% Trition X-100, and counterstained in PBS containing 6.6nM ALEXA647 phalloidin and 10nM DAPI for 30 minutes. The samples were then washed four times for 5 minutes with PBS. Coverslips were mounted onto slides with ProLong Glass Antifade and allowed to dry. Images were obtained using an Olympus IX-83 microscope with a 60x/1.4NA objective, Sutter shutters & filter wheels, Sedat quad filter set (Chroma), and a Hamamatsu ORCA Flash 4v2 camera. The microscopy hardware and image acquisition settings were controlled by Metamorph software. Typical exposures times were 200ms for ALEXA647 phalloidin and 300ms for GFP and mcherry fluorescence channels. Exposures ranged between 2-10ms for DAPI. Images were collected in all four channels for all treatment groups.

*Tip to cell body ratio, filopodia density quantification, and analysis*

FIJI was used for image analysis (Schindelin et al., 2012). The ratio of tip intensity to cell body intensity (TCBR) was calculated by using 4x4 pixel regions-of-interest to determine the intensity of the background adjacent to the filopodium (I _b_ ), the filopodial tip (I _t_ ), and the cell body (I _cb_ ). The ratio of the I _t_ to I _cb_ was calculated after subtracting I _b_ from those measurements using the equation TCBR = (I _t_ -I _b_ )/(I _cb_ -I _b_ ).

Filopodia edge density was calculated from regions of a transfected cell's periphery not in contact with other cells (free cell edge). Lengths of free cell edge were measured and the number of filopodia along those lengths were counted manually. Filopodia edge density is then calculated as the number of filopodia divided by the length of the measured free cell edge.

Mean cell brightness was not significantly different between any of the mchr-tagged constructs, nor was mean cell brightness significantly different between any of the GFP-tagged constructs used in this study (Phan and Quintero-Carmona, 2026).

*Phyre modeling*

To predict the tertiary structure of MYO3A, amino acids 316-1616 of the human MYO3A sequence were submitted into the Protein Homology/analogy Recognition Engine (Phyre2.2) web server. Given the sequence, the engine uses homology identification and secondary structure prediction to determine structural homologues by a profile-profile alignment algorithm (Powell et al., 2025). High scoring alignments are then used to construct a proposed tertiary structure of the submitted sequence by comparison to structural databases. Alternatively, unknown structures can be fit directly to a specific, known structure, which was our approach. As the regions of myosins directly involved in the actin-binding interface were not well characterized in structures generated by X-ray crystallography or cryo-electron microscopy at the time that these studies were initially undertaken (Lorenz & Holmes, 2010), Phyre2 was used to predict the structure of the actin-interface of MYO3A through aligning the sequence of human MYO3A directly to the model of the actomyosin interface generated by Lorenz and Holmes (Lorenz & Holmes, 2010).

*Quantification and statistics*

Each condition of each cell-based assay was repeated independently 5 to 8 times--a biological replicate represented as "n". For each experimental condition, multiple measurements were averaged for each day with each day serving as a biological replicate. For TCBR, each biological replicate consisted of at least 10 cells with no more than 5 filopodia assayed per cell. At least 7 cells were measured for each biological replicate for filopodia edge density.

Statistical significance was determined by ANOVA followed by a Tukey test based on the set of biological replicates for each condition. Kaleidagraph 4.5 was used to calculate descriptive statistics and carry out the ANOVA with Tukey analysis. Quantification presented in the text are written as mean standard error of the mean.

*Selection and preparation of sample images for publication*

Sample images chosen for the figure were chosen as representations of the patterns observed in the quantified data. The software packages Metamorph, and FIJI were used to generate the pseudo-colored images included in the figures.
